# Discernment on assistive technology for the care and support requirements of older adults and differently-abled individuals

**DOI:** 10.3389/fpubh.2022.1030656

**Published:** 2023-01-09

**Authors:** P. Muthu, Yongqi Tan, S. Latha, Samiappan Dhanalakshmi, Khin Wee Lai, Xiang Wu

**Affiliations:** ^1^Department of Biomedical Engineering, SRM Institute of Science and Technology, Chennai, India; ^2^The 71st Group Military Hospital of the Chinese People's Liberation Army, Xuzhou, Jiangsu, China; ^3^Department of Electronics and Communication Engineering, SRM Institute of Science and Technology, Chennai, India; ^4^Department of Biomedical Engineering, Faculty of Engineering, Universiti Malaya, Kuala Lumpur, Malaysia; ^5^School of Medical Information Engineering, Xuzhou Medical University, Xuzhou, China

**Keywords:** assistive technologies, intellectual disabilities, machine learning, telecare, autism

## Abstract

Assistive technology for the differently abled and older adults has made remarkable achievements in providing rehabilitative, adaptive, and assistive devices. It provides huge assistance for people with physical impairments to lead a better self-reliant daily life, in terms of mobility, education, rehabilitation, etc. This technology ranges from simple hand-held devices to complex robotic accessories which promote the individual's independence. This study aimed at identifying the assistance required by differently-abled individuals, and the solutions proposed by different researchers, and reviewed their merits and demerits. It provides a detailed discussion on the state of art assistive technologies, their applications, challenges, types, and their usage for rehabilitation. The study also identifies different unexplored research areas related to assistive technology that can improve the daily life of individuals and advance the field. Despite their high usage, assistive technologies have some limitations which have been briefly described in the study. This review, therefore, can help understand the utilization, and pros and cons of assistive devices in rehabilitation engineering and assistive technologies.

## 1. Introduction

Assistive technologies are used by individuals with special needs which help them in performing their daily routine or accessing computers for self-care activities. Globally, billions of people need assistive products but they don't have access to them. Wheelchairs, spectacles, prostheses, tablet organizers, memory aids, etc., are examples of assistive products. Advancements in assistive products help persons who need them to come out of their isolation, reducing their dependence on others for their health and care, and building confidence in them. People with non-communicable diseases like stroke or diabetes, persons with mental health conditions like autism or dementia, individuals with slow health deterioration, and older adults need assistive technologies for their positive wellbeing and health.

Affordable assistive technologies are needed for people across low-income countries. Wheelchair access is required by nearly 75 million people, but only 10% of them have it. Of the 460 million people with hearing challenges, only 10% have hearing aids. Most persons with poor vision cannot access assistive devices to correct their vision. The development of affordable, easily available, non-invasive assistive devices will help those in need to lead a fully functional life (1).

**Figure 1:**
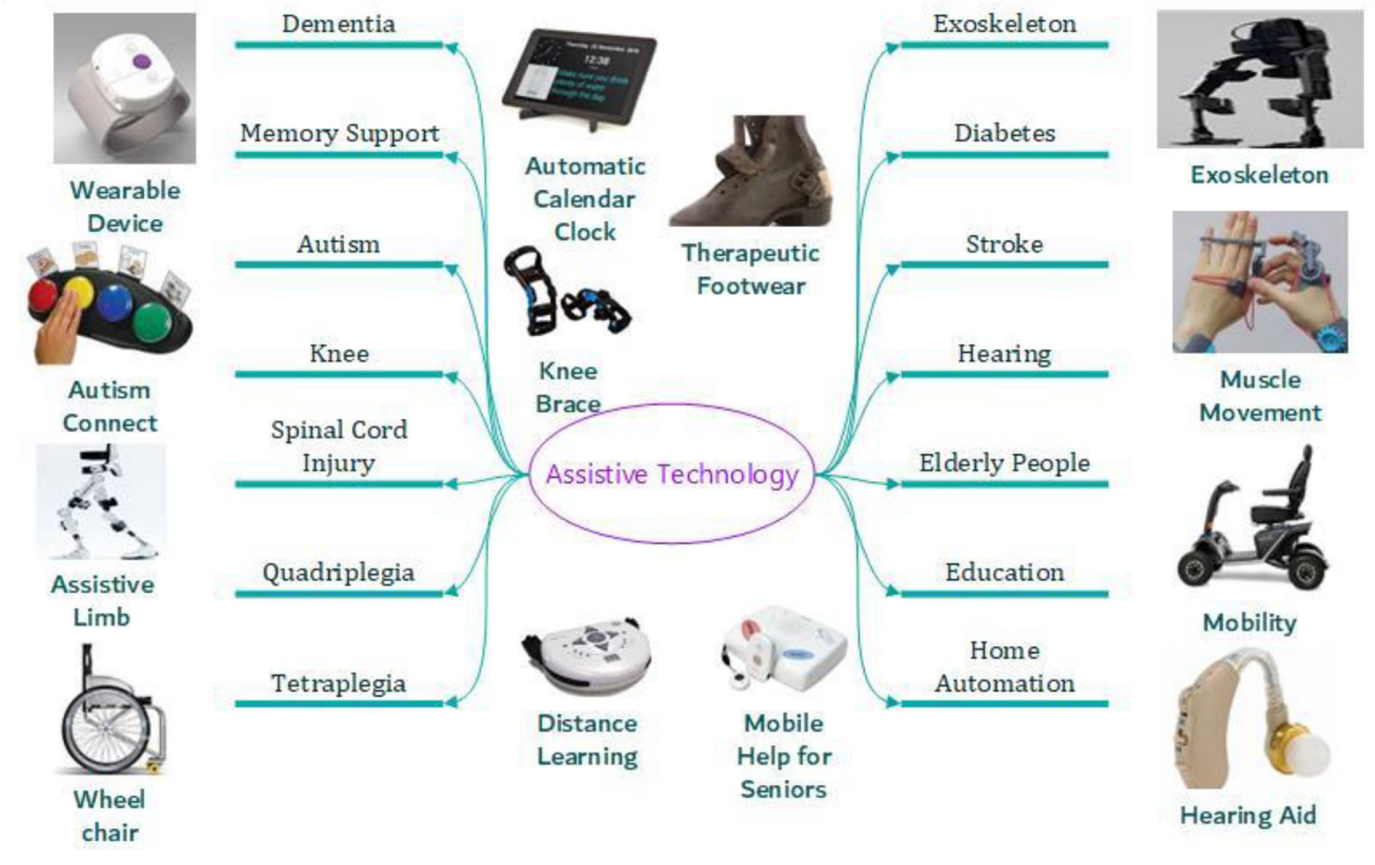
Assistive technology application fields.

Assistive technology has wide applications in multiple fields of individual care and is not limited to mental health. Its use is evident in dementia (assistive technology for memory support), autism, knee/spinal cord injury, quadriplegia, tetraplegia, exoskeleton, diabetes (therapeutic footwear), stroke, mobility, cognitive, visual, eating, hearing impairments, personal emergency response systems, accessibility software in education, computer accessibility, home automation, walkers, elderly care, IT accessibility, etc. Figure 1 offers some examples of application fields where assistive technology is used.

Assistive devices can also be used for individuals with gradual functional decline, orthotics, vehicle modifications, recreational aids, environmental controls, communication, assistance dogs, and learning. Diagnosing the impairments and assisting in the proper use of assistive technology can be made possible using artificial intelligence and machine learning techniques for more accurate diagnosis (2). The related apps and smartphones have made the process of applying assistive technologies easier, making it more user-friendly for the common man. This paper reviews assistive technology for individual care and the related work in literature.

### 1.1. Review outline

The following contributions are made through this review study:

(i) A detailed discussion on the available assistive devices for differently-abled individuals and older adults reviews their merits and demerits and provides key insights regarding their usage. This review includes rehabilitation research, which previous studies have overlooked.
(ii) A systematic review of assistive devices and their usage in various application fields of assistive technology is undertaken.
(iii) A comprehensive discussion reviews the challenges in using these devices for the differently-abled and offers suggestions on how they can be overcome.
(iv) Future research pathways relating to individual care using assistive devices for learning and computer accessibility are analyzed.
(v) This review emphasizes how most of the research on assistive devices can help advance research on other diseases.

## 2. Application fields of assistive technology

Robots-assisted surgery and AI-powered medical devices prove the emergence of machine learning (ML) and AI techniques in assistive technology, with doctors and entrepreneurs paving the path for an AI-based future. Regardless of an individual's ability, effective implementation of assistive technology improves the person's accessibility and quality of life. The application fields where assistive technology is used and applied are discussed below.

### 2.1. Mental health—Dementia

Dementia is caused by many diseases, which mainly affect the thinking, memory, and social abilities of the individual. Disorientation, confusion, poor motor functions, planning, organizing special needs, complex task handling, problem-solving, reasoning, communication issues, and memory loss are the cognitive changes caused because of dementia. Damage or loss of nerve cells and their brain connection is the cause of dementia. The different types of progressive dementia include vascular, frontotemporal, Lewy body, Alzheimer's, and mixed dementia. The individual may lack social behavior, language, memory, and attention. Table 1 provides an overview of recent research in dementia care.

**Table 1:** Summary of previous research on dementia care.

**Reference**	**Model**	**Performance**	**Dataset**	**Influencing factors**
Shuai et al. (3)	7 features were selected for predictive model. kNN proved efficient	Accuracy: 0.84 ± 0.0242	Data collected from 40 participants	The person living alone or not is also considered for study
Suijkerbuijk et al. (4)	Personal evaluation game—to evaluate the sleeping pattern of individual	First-hand experience data obtained	Data collected from 3 dementia patients. 26 photos, 3 s audio were gathered	425 answers received. Tablets, laptops, paper versions used
Sonja et al. (5)	Binary logistic regression model	82.5% accuracy; 0.89 sensitivity and 0.64 specificity	40 individuals data were collected from patient visit logs and databases	Four fold validation was performed. Adopters and non-adopters were identified
Juan et al. (6)	Mobile app for daily tasks micro-prompting, outdoor, indoor way finding,	Quantitative study results after hands on experience	NA	Studies carried out in ambient intelligence lab were discussed
Bruce et al. (7)	Game to identify cognitive ability changes—regression to measure related features	Session score level gave 1 false positive and 1 false negative for eleven participants	12 participants from adult day program joined the study	Minimental state exam score was considered based on their demographics

Shuai et al. proposed a predictive model for use of assistive technology by individuals with dementia. A video streaming system using a mobile phone was developed with data mining approaches for classification. The streaming system provided reminders for everyday tasks, which is helpful for the individual and the caregivers. The extracted data included details such as gender, mini-mental state examination, age of the individual, patient's equipment skill, previous profession details, mobile reception, broadband, existing preparation, caregiver involvement, physical health, and extra support. A greedy algorithm step-wise regression was applied to select the best features. CART decision tree, KNN, adaptive boosting, support vector machine, decision tree, Naive Bayes, and neural network algorithms were applied for classification. K nearest neighbor (KNN) with seven features provided an accuracy of 0.84 ± 0.0242 (3). Additional data and more work on caregivers are required for improving the prediction model.

Suijkerbuijk et al. proposed a suitable private assessment model (designed in the form of a game) to appraise assistive technology for dementia-recovered individuals for their daily assistance. Twelve households having persons with dementia supported by a caregiver participated in the study to provide a first-person perspective for the research. The developed personal evaluation game included an ecologically valid tool that was explorative and qualitative. Options to take pictures, speak, and write were included in the game since the person's preferences were unknown. More options were included to facilitate longitudinal and flexible data collection to provide providing complete insights into the use of assistive technology for the person. The proposed game, “Aangenaam,” collected data on experiences related to day-to-day happenings, personal events and targets, and physical and social context through flexible choice-based question cards. The question cards had intelligent, simple, personal, environment, and daily activities interactive questions.

A likely drawback of the proposed game is that the caregivers' involvement may potentially provide biased and less valid answers (4).

In another study, Sonja et al. (5) developed a usage-prediction tool and technology adoption for assistive technology for persons with dementia. Forty participants were included in the study and the parameters considered were MMSE, age, gender, previous profession, physical health impairments, broadband connection information, technology knowledge, caretaker participation, mobile phone usage, extra support by friends and family members, adoption level, alleged utility and caregiver's gender, and age. A binary logistic regression model was used for classification after factor analysis and assenting classification assessment. In their study, Juan et al. (6) provided a hands-on experience for persons with intellectual ailments using assistive technology and discussed its challenges and opportunities.

Bruce et al. proposed a modified “whack-a-mole” game for individuals with moderate dementia to identify intellectual capacity variations. Twelve adults with dementia were studied for 1 year using a mobile app game (7). Session score levels were measured and it proved on day progression.

### 2.2. Mental health—Autism

Autism is a neurodevelopmental disorder characterized by repetitive behavior and challenges in social communication. Affected persons find it difficult to express themselves and are unable to understand other people's thoughts and feelings. While they lag in some skills, they can develop skills like problem-solving and analysis. The types of disorder manifest in autism are Asperger's syndrome, pervasive developmental, childhood disintegrative, and autistic disorder. Autism levels are determined by support requirements at various levels and development assessments and constant evaluations can help in the early diagnosis of autism in children. The different levels of autism are as follows.

1. Asperger's Syndrome or level 1 spectrum is where the individual possesses good verbal skills and intelligence but faces problems in social communication.
2. Rett Syndrome affects the child's life, but family support can help them.
3. Childhood Disintegrative Disorder, which causes delayed development in social, language, and motor skills.
4. Kanner's Syndrome is a classic autistic disorder. The individual is alert, attentive, and intelligent but possesses issues in interaction, holding things, etc.
5. Pervasive Developmental Disorder—Not Otherwise Specified (PDD-NOS), where the individual exhibits mild autistic behavior.

Based on the level of autism, diet and behavioral modifications can help the individual. Technology helps children with autism to improve in multitasking. But digital technology makes the individual focus in one task at a time. There are app-based assistive devices that are currently available for autistic individuals. They are necessary for their development. But it is additional work for the caregivers/parents to minimize their usage and ensure that their wards do not become too dependent on assistive devices.

For people with autism, assistive technology can be used to improve behavior, academic skills, cognitive development, social skills, and communication. Some examples of assistive technology aids include social stories, social rule cards, social scripts, audio, videotaping, schedules, forewarning, directions, digitized speech, pencil augmentations, pencil replacements, and computer-based and tablet-based devices.

Noreen et al. examined the use of robots to improve learning and cognitive abilities in children with autism. LEGO Mindstorms EV3 robot was used for eight children and provided positive improvement in learning among them. It created a happy learning environment, engagement, interest, focus, attention, communication, and interactions (8) by adopting non-formal therapy and learning, supporting autism in early years, reinforcement cycle, action, and integrated activities with motor manipulation. However, not every school or person can afford to buy the robot, which is a limitation. One session with limited period access was given to each child which was not sufficient for the study. In addition, the study population was too small.

Along similar lines, Katrin et al. (9) reviewed robot-based intervention for persons with autism. The study participants were categorized based on age, gender, and wordings for participants. The robots were used for skill development like emotion, expression control, recognition, social convention, interaction and responding. The recommendations of the review were based on longitudinal studies, course of intervention sessions, study design, participants' experience with the robots, participants' characteristics, a measure of engagement, robot types, and supporting members assignation while in the study. However, further research is required, particularly in robot-based interactions for autism affected children with a greater number of samples.

Michal et al. proposed a stress-observing device for persons with autism. A wristband comprising autonomic wearable components with mechanical, electronic parts, and software measured movement, temperature, heart rate, and skin resistance using sensors (10). Stress results in neuro-hormonal modifications in a flight-or-fight state. Therefore, the stress data of the individual was recorded over time on a device that was easy to use, flexible, and wearable. The device was helpful for therapists and educational institutions having individuals with autism. Similarly, Uttama et al. (11) designed an adaptive gaze-sensitive response technology for children with autism using virtual reality (VR). The real-time observing patterns helped quantify a person's engagement level and improve the children's social communication behavior. The device, composed of a VR-based task presentation module and a bi-directional conversation module design, included an adaptive response unit with performance-sensitive and engagement-sensitive components. The limitation of the wearable device is that it used a bidirectional conversational module and wearable eye tracker which is not suitable for less-functioning individuals with autism.

**Figure 2:**
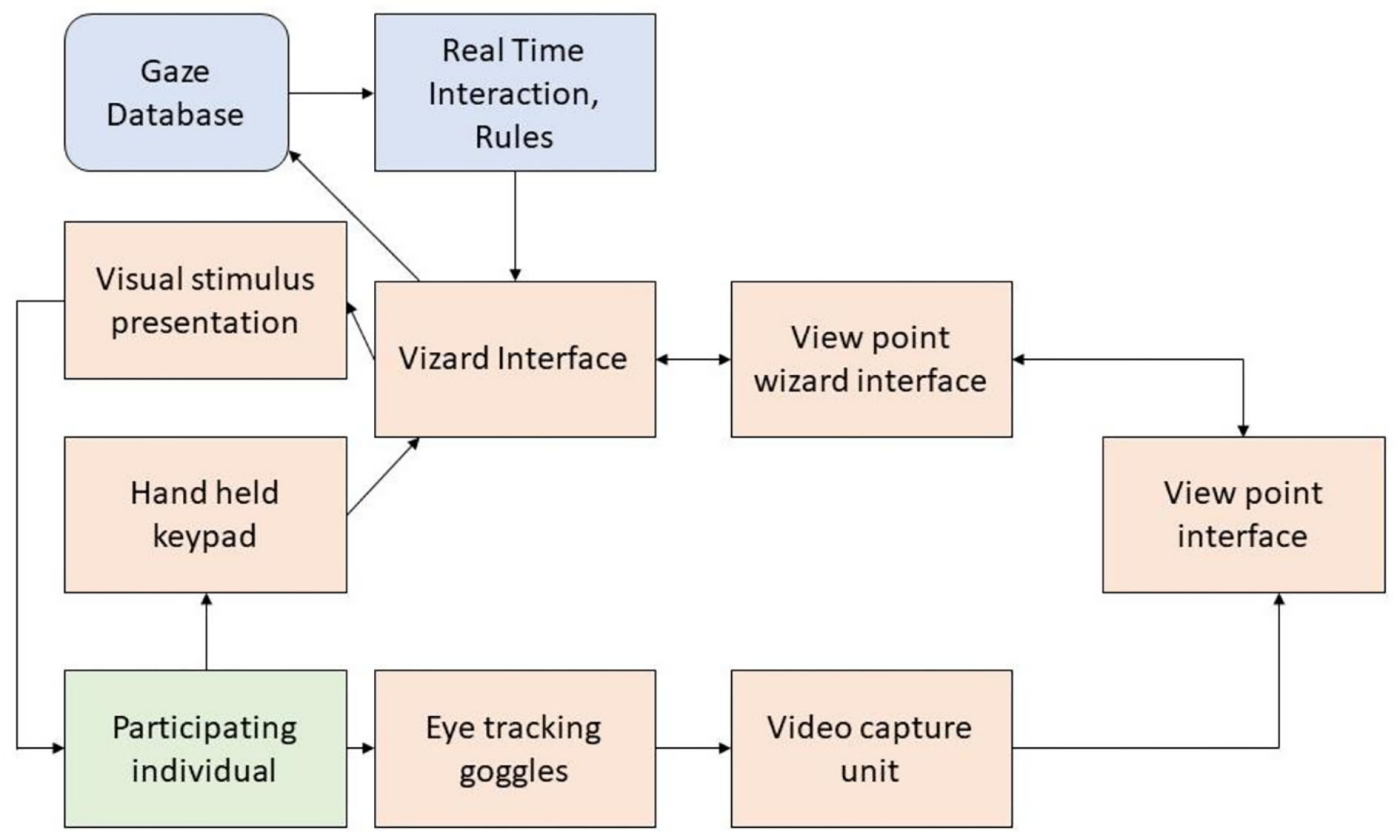
Data acquisition and control mechanism of the VR-based eye-gaze monitoring system.

Uttama et al. (12) also designed a gaze-aware virtual social interactive system for children with autism. People follow different viewing patterns while communicating, so analyzing the eye gaze can offer a few indications. In this interactive system, a story is created in virtual reality (VR) using visual objects called avatars, with neutral, angry, and happy facial expressions. While interacting with the VR avatar, the individual's eye gaze is monitored. The data acquisition and control mechanism followed is given in Figure 2. The work-related event identifiers are stored in the gaze database. The eye tracker records the user's eye movement using a video recorder with Viewpoint software. The features extracted from the database are the mean blink rate, pupil diameter, average fixation period, and total fixation counts. Real-time feedback is provided to the user based on the acquired data and analysis.

In their study, Fabio et al. explored applied behavior analysis for treating autism by reviewing 86 articles and offering their insights. Persons with autism may be categorized as severe, moderate, or mild. The study analyzed the behavioral studies that were done in past but found no data to ascertain whether the observed behaviors were socially significant. In addition, the targeted behavior studies were incomplete because of limited data availability. The studies presented more user test results but their acceptance and validations were not included. Robots helped in motivating persons with autism to undertake repetitive and specific tasks (13). Web systems based on robots, image processing, gamification was developed for autism affected children. Cognitive ability of the autism individuals was different from those with other mental disabilities.

The features extracted from the system are the sum of fixation counts, the average rate of blinking, the rate of fixation, the average fixation from each segment of the monitored signal, and the average pupil diameter. The unit was tested with six adolescents for engagement measurement.

### 2.3. Knee replacement

Knee arthroplasty or knee replacement surgery is required when the knee joint is damaged reducing mobility and causing extreme pain in patients. It can be caused by hemophilia, osteoarthritis, unusual bone growth resulting from disorders, injury in the knee, loss of cartilage resulting in knee deformity, blood supply issues causing the death of bone in the knee joint, or gout. The amputation rate caused by an infection resulting from a knee replacement is 0.1–10%. Knee replacement surgeries may be total or partial replacement procedures and some of the available assistive devices for knee replacement are folding shower chairs, quad, clip and pulling dressing aids, walkers, abduction pillows, and raised toilet seats (14).

Li Zhang et al. (15) reviewed assistive devices for the human knee joint based on mechanical system design components such as actuation, joint alignment, human attachment, and power transmission design. The control systems and sensing design were based on human device interaction signals, control system-based device signals, and control systems dependent on human manual signals. However, the actuator devices were bulky, stiff, consumed more energy, and heavy. Wearing comfort and good human device interaction with soft and passive actuators are suitable assistive devices.

For gait rehabilitation, Hao et al. proposed a user-dependent aid made of assistive knee braces. The brace was designed by configuring the appropriate stages of various assistive functions using a fuzzy expert system for gait analysis and reviewing the patient's physical condition (16). A reference key trajectory was generated based on the individual's gait and the force was measured using a cross-impedance controller. The objectives of the assistive functions were torque assistance and trajectory control (17).

In their study, Siyu et al. suggested a wearable knee assistive device for construction workers' kneeling tasks. The integrated inertial measurement units estimated lower limb pose and gait (18). These data can control the assistive torque of the knee joint with quasi-direct drive propulsion. The knee exoskeleton included a waist belt, shank support frame, loadcell, actuator, thigh support frame, elastic straps, hinge, linkage, and control electronics. The beginning and finishing of the kneeling gait were identified by a threshold-dependent kneeling gait recognition process (19). Comfort and fit on wearing the device had to be taken care of. A half-rigid assistive device for the knee exomuscle was developed by Zhang et al. (20) which used the advantages of soft and rigid structures. A route was developed for the tendon and the system removed the misalignment between the center of rotation of the knee joint and the device. Deterministic load compensation was achieved with 38 Nm assistive torque to the knee joint.

**Figure 3:**
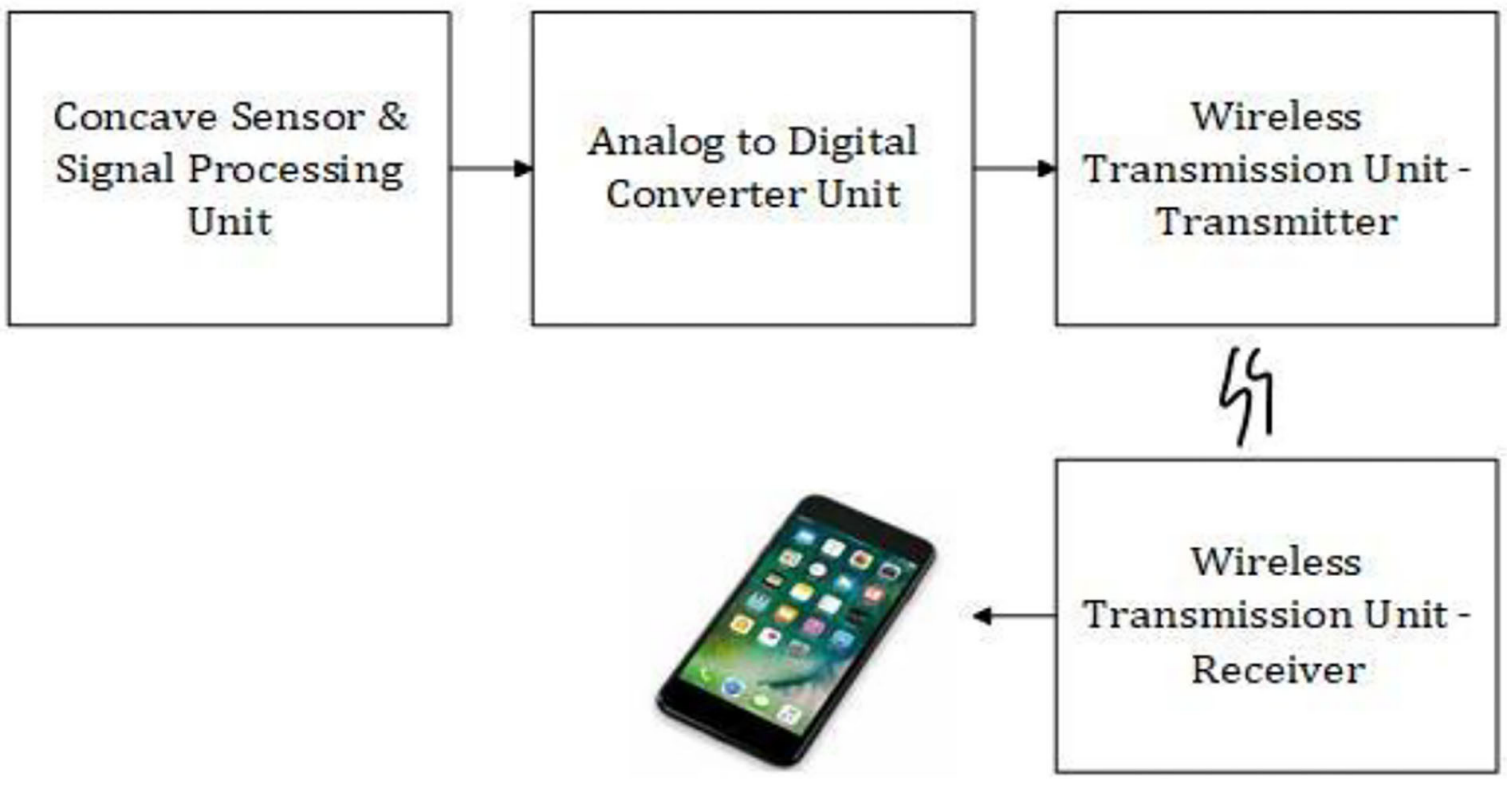
System design of the wearable pulse oximeter device.

A wrist pulse-oximeter was developed by Grantham et al. (21) to find pulse rate and oxygen saturation level in an individual's blood which is of neo-reflective type. The data was wirelessly transmitted to a mobile phone for analysis. The system design is given in Figure 3.

Signal processing was done by low pass filters to acquire DC components, band pass filter, and amplifier to obtain the amplified AC component from the concave sensor module. The AD converter converted the signal to a digital component so that it can be easily sent to wireless modules and processed. The wristband and the smartphone are connected wirelessly with a transmitter and receiver.

Massimo et al. (22) modeled the human knee for assistive technologies using motion capture technology and an EMG-based musculoskeletal knee model. They proposed a muscle model based on infinitely stiff tendons which could speed up the muscle force computation and joint movement calculation accurately and 3D musculo tendon kinematics were derived from the model. The process included gathering human motion information by movement capture method, modeling and simulation of the logged human motion, and setting and implementing the EMG-driven model.

Using assistive control models, Mohammad et al. (23) proposed a motion estimation for the lower limb using ultrasound imaging. The rectus femoris muscle's ultrasound features were analyzed while on a non-weight-bearing extension trial. A random sample consensus (RANSAC) algorithm was applied for the segmentation of the muscle and fascicle after a multi-scale ridge filtering to excerpt the structures. The model parameters were estimated, though outliers and noise were presented by the RANSAC, in a two-phase hypothesis generation and evaluation technique. MSAC (m-estimator sample consensus), a modified version of RANSAC, could detect accurately even in the presence of other objects. Weights for cost function evolved from the grayscale intensities (24), and the angular velocity and the knee joint angle were found with an average root mean square error of 0.262 rad/s and 7.45°. However, deformation may lead to modifications in muscle morphology, resulting in a blurry image and reducing the accuracy of segmentation.

### 2.4. Spinal cord injury

Disks, ligaments, and vertebrate damage lead to spinal cord injury causing dislocation, spine fracture, and compressing or crushing of vertebrates. The related complications are bowel, bladder, and circulatory control, pressure injuries, breathing difficulties, bone density issues, depression, pain, sexual health, wellness, fitness, and muscle tone problems. Minimized functional movements and activity limitations at different levels suggest the need for assistive devices for individuals with spinal cord injuries. These assistive devices include wheelchairs, hand bikes, canes, crutches, adapted shoes, walking frames, full and semi-electric or manual beds, transfer boards, positioning devices, ventilators, etc.

Replacing a missing body part to perform the part's tasks is called a prosthesis. Neuroprosthetic devices can substitute for impairments in sensory, motor, or cognitive functions caused because of nervous disorders. These types of prosthetics could be transradial, transtibial, transhumeral, or transfemoral. Based on the specific needs of the person, full or partial prosthetic hands or feet can be replaced. Artificial fingers, noses, and eyes are also available.

Rudiger et al. (25) proposed a non-invasive hybrid neuroprosthesis to be used after spinal cord injury of paralyzed upper extremity functional rehabilitation. A hybrid system comprising orthoses and functional electrical stimulation can restore a fully lost arm tasking. Interfacing the brain and computer depending on motor images is an excellent alternative instead of a neuroprosthetic regulator. Lateral grasp helps in lifting flat things between flexed thumb and fingers, and palmar grasp positions the thumb in opposition to the index finger allowing easy handling of larger objects generated by grasp neuroprosthesis. The hybrid system was evaluated with performance metrics reliability, stability, delay, degrees of freedom, selectivity, and the number of discrete levels of activation.

For individuals with spinal cord injuries and muscle interfacing implantation, an intramuscular EMG- based musco-skeletal device was proposed by Jung et al. (26). Joint torques are estimated to control assistive devices with entrenched EMG sensors. There exists a non-linear relation between muscle activation and neural activation given by:


(1) $$\begin{array}{l} m\left(t\right)=\frac{e^{Su\left(t\right)}-1}{e^{S}-1} \end{array}$$

where *m*(*t*) is muscle activation and *S* is a non-linear shape factor, inside the range (−3, 0). The best fit normalization is body weight times height joint torque normalization, which assists in analyzing the effect of weight and height on peak joint torque values.

**Figure 4:**
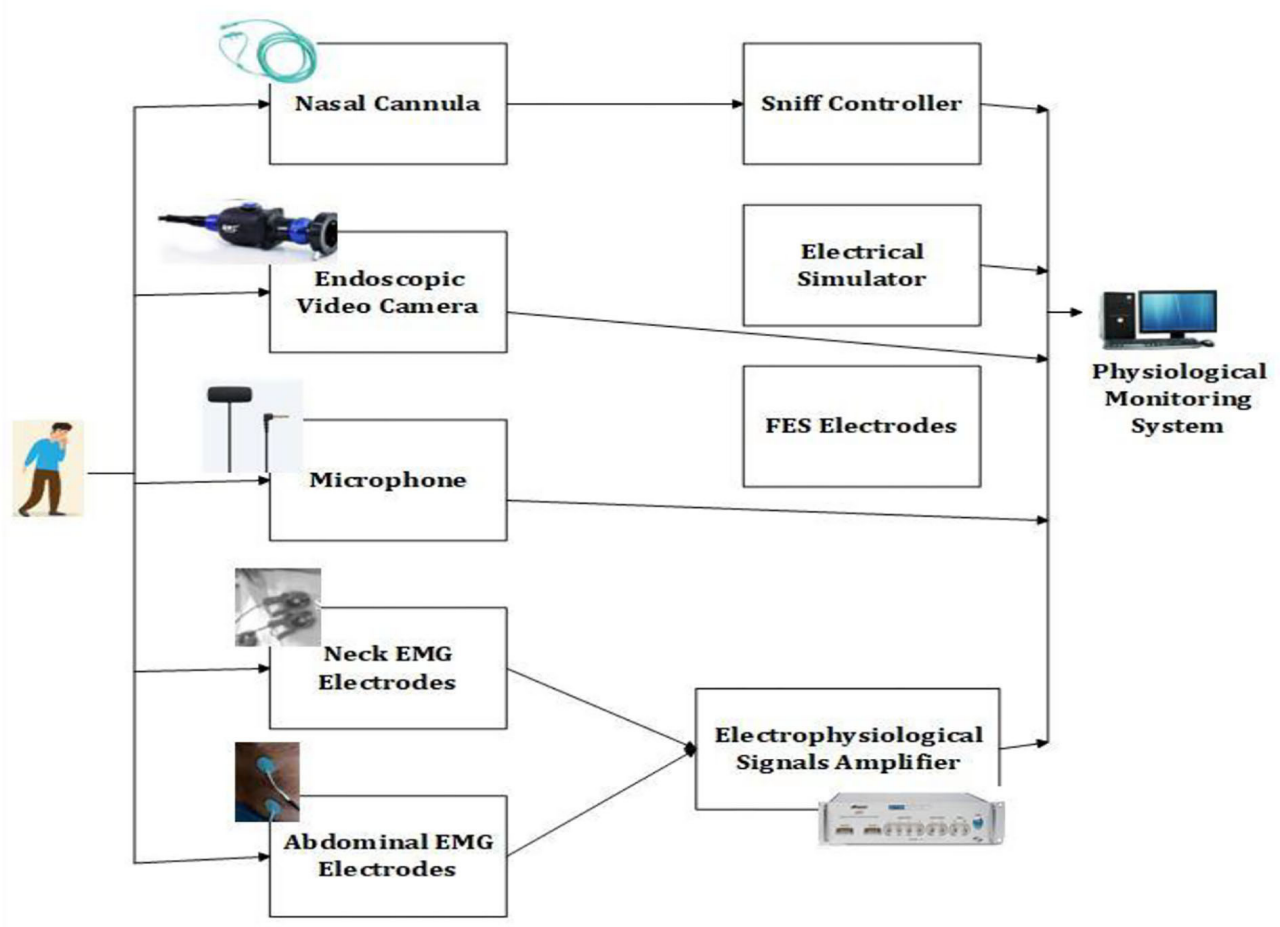
Experimental setup for cough stimulation.

Patients with cervical spinal cord lesions followed by coughing require assistance and in their study, Lior et al. (27) propose a sniff controller to self-help stomach functional electrical stimulus. Through changes in nasal airflow, the sniff controller can trigger peripheral devices. Slight changes in the nasal airflow are converted into electrical signals that can control exterior devices. It can control assistive devices for communication to mobility. The Functional Electric Stimulation (FES) is self-triggered by the sniff controller. The activated device could increase peak exploratory flow ±25–27%. The device setup had the following components as shown in Figure 4. The endoscopic video camera, microphone, nasal airflow measuring device, and signal amplifier are synchronized. Eight electrodes are connected with two sets of output poles for acquiring the data.

A mini wireless nasal sensor instead of a nasal cannula can be an optimal sniff-controlled system. The nasal cannula is a device through which supplemental oxygen therapy is delivered for individuals with lower oxygen levels, and can be used for low or high flow. It is used until the patient recovers from the illness and is replaced every 3 weeks of use.

Dae et al. (28) undertook a pilot study to identify the use of wheelchair-mounted robotic arms for spinal cord-injured patients. Two control modes were set, and performance was measured for baseline characteristics, psychometrics, and quantitative performance measures. Learning improvement by minimizing command count and mean task completion time was seen in the manual mode and variability minimization was seen in the auto mode. The study was conducted over 3 weeks and the steps involved pre-evaluation, initial training, top-shelf, bottom-shelf, and final training followed by post-evaluation. This assistive device could improve the pick and place tasks of the patients involving two shelf levels and six objects.

### 2.5. Exoskeleton

The biological capacities of humans can be enhanced to a greater extent by wearing an external frame support called an exoskeleton. It also helps to overcome injuries caused. A lower limb exoskeleton helps individuals when they are not able to perform their daily actions. They aid walking and work using electric motors. The upper limb exoskeleton is a wearable device joined to the arm. Signals to control the device are given by the user (29). Active exoskeletons are those with motors and passive ones are those without motors. The risk of musculoskeletal injuries can be reduced while lifting heavy objects using a powered or active exoskeleton. Passive exoskeletons are used to hold objects, provide ergonomic support, and prevent injuries. Quasi-passive exoskeletons are used to carry loads while walking and include a knee variable damper, and hip and ankle springs.

The exoskeleton gloves include actuators and sensors to mimic human muscles and nerves. Sarac et al. (30) surveyed hand exoskeletons for assistive, haptic applications, and rehabilitation. The treatment process and motor learning were significantly improved by using assistive technology during physical therapy sessions. A wearable hand exoskeleton gives accurate kinesthetic feedback through active force transmission using mechanical components. The features required from the device are safety, hand anatomy, affordability, comfort, and effective force transmission. Assistive exoskeletons help differently-abled persons to grasp and hold objects. For wider usage, the exoskeletons should be easily affordable, natural, wearable, lightweight, and portable.

Akim et al. (31) explored the application of assistive-powered upper limb exoskeletons, especially for older adults for performing their daily tasks. Beginning from the first prototype for upper limbs where the hand was attached to a string and modifying the string length exerted arm movement and the motor and control parts were located on the backside of the wheelchair, the authors review various prototypes. In the latest exoskeleton under research (AXO-SUIT) the user-robot interaction is carried out through the force sensors dispersed in the upper limbs. The limb movement at the joints is assisted by the actuators. Electric actuators providing the highest precision for position control, easy programming, silent operation, smooth functioning, and high repeatability were used with trade-offs in cost, size, and weight. Sensors that could analyze limb motions improved the interaction feedback between the exoskeleton and the user.

Wujing et al. (32) designed and tested a hip assistance soft exoskeleton with hip flexion, hip extension, and flexion assistance modes. A feedforward model controlled the assistive force with a proportional derivative reiterative learning controller. The proposed exoskeleton was analyzed during the walking motions. Based on metabolic reduction, the hip extension model recorded improved performance compared to hip flexion for single hip movement assistance.

**Figure 5:**
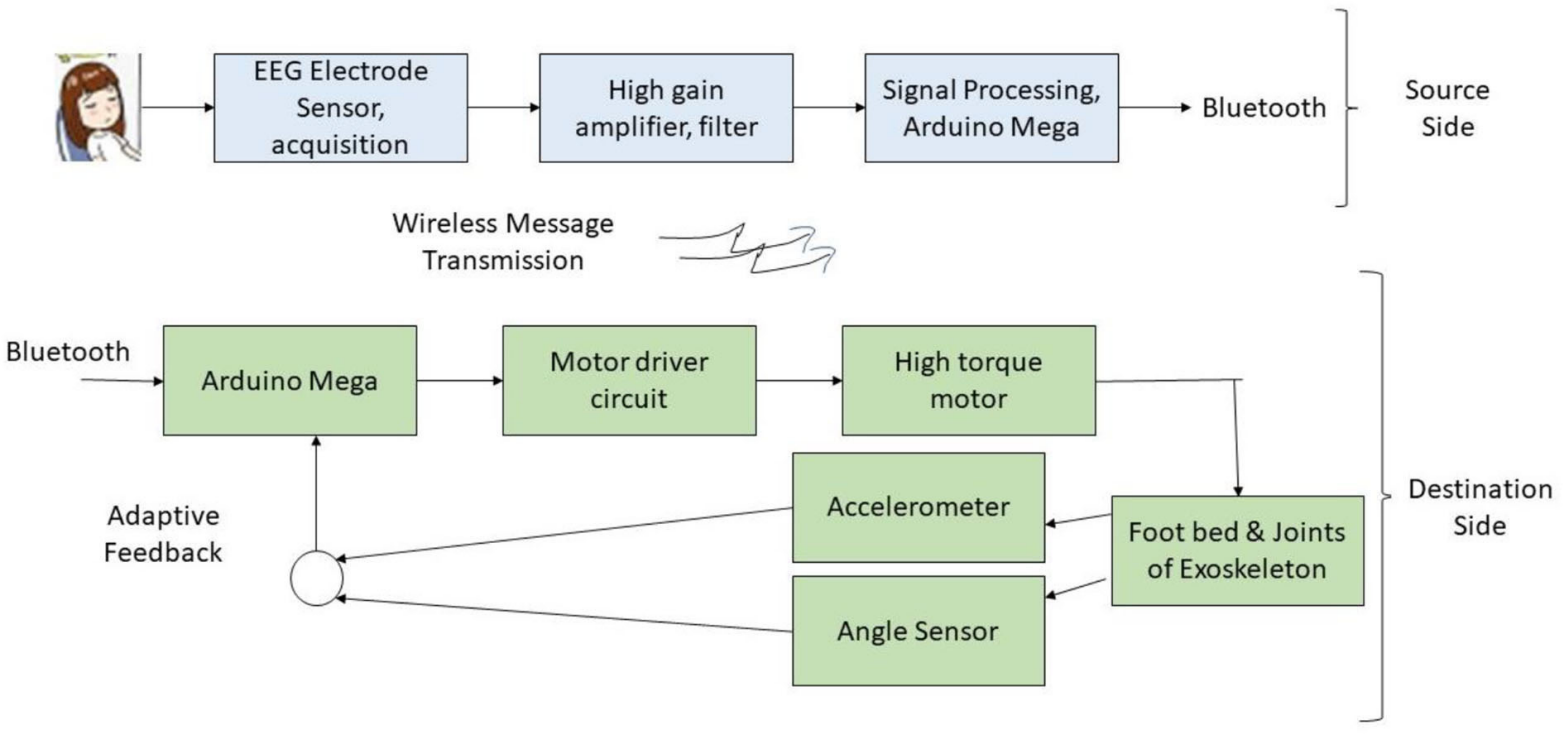
Experimental setup of the brain-activated multi-dimensional exoskeleton.

Rehabilitation of post-stroke paralyzed patients through a brain-assisted adaptive lower limb exoskeleton is discussed by Vinoj et al. (33). The false rate of the device was minimized through adaptive sensory feedback. Impairment-based flexibility, emergency data transmission to caregivers, and a microprocessor-based brain computer interfacing system for signal transmission are some of the features of the exoskeleton which achieved a classification accuracy of 80%. A threshold for tilt is inbuilt to identify the fall of the patient and the caregivers are called for emergency rescue. Figure 5 illustrates the experimental architecture of the proposed brain-actuated exoskeleton, which had separate source and destination sides, and the human controls the exoskeleton. The electroencephalograph (EEG) signals are obtained by non-invasive method from the person. A set of fourteen electrodes calculate the measurement, including two reference electrodes. The signals are amplified and filtered for analysis (34). After windowing, the data is converted to digital and given as input to the microcontroller. The controller learns the data after extracting their features, for the commands right and left turn, sit, stand, and forward motion. Machine-learning-based training and testing were performed. Connection to the exoskeleton is through Bluetooth and the controller converts the results and motor action. Regular feedback is obtained for further modifications.

Walsh Hadamard transform extracts the user motion features to be sent to the lower limb and brain through Bluetooth. The extracted features and Hadamard coefficients are then used for actual brain signal reconstruction (35). However, the inconvenience of wearing a headset and the high cost of the device are trade-offs of the device.

Patane et al. proposed a compliant lower limb multi-joint exoskeleton for pediatric patients with neural problems with their locomotion and ankle-knee motion. The wearable device with a position control worked in coordination with the individual's musculoskeletal system. 5.6 Nm/rad torque and 8.8% hysteresis were achieved (36). Walking assistance testing was performed, and the device assisted in the right foot landing in the gait cycle start. Heel strike, swing, mid and fore strike features were used for the device operation.

### 2.6. Diabetes—Therapeutic footwear

Insulin pens, syringes, pumps, sugar testing devices, strips, diabetes medical alert bracelets, etc. are the devices necessary for individuals with diabetes. Peak plantar pressure is found in older adults with diabetic neuropathy and therapeutic footwear is more efficient in making them walk comfortably. Lesions are not felt because of sensory damage, resulting in ulcers (37). The peak plantar value of 599.9 g/cm^2^ on barefoot decreased by 4.3% using normal footwear; wearing diabetic footwear recorded a reduction of 22%. The use of therapeutic footwear has been reviewed by Gustav et al. (38) in their study.

### 2.7. Stroke

Stroke may lead to paralysis of the body on one side or weakness, alterations in understanding, learning, and speaking capabilities. Assistive technology for stroke-affected people includes live scribe echo smartpen, shower chair, Velcro fastenings, pocket talker, foot socker, echo dot, etc. Zih et al. (39) developed a 3-dimensional printed multi-functional hand device for assisting persons affected by stroke to grasp objects. They found that the hand clutch strength improved by 36% and adjacent weak energy by 54.1%. Biomechanical evaluations were performed in terms of hand function, strength, and statistical analysis. The device also improved the training frequency, rate of recovery, and level of stroke patients. Lateral pinch and grip force improvements were clearly visible.

Huang et al. (40) proposed a similar device for coordinated functional opposition for advanced prehensile motion and manual dexterity. Following informed consent, a baseline assessment was undertaken, and randomization was performed for a study population of ten. Measurement, manufacturing, and task-oriented training of the device were executed. Post-term and follow-up assessments were done to analyze the device's operation. A portable assistive glove for hand-impaired stroke patients was proposed by Heidi al. (41). Passive cyclical stretching with active assisted task-dependent training improved doing tasks and upper limit motor control. The components included a control box, linear actuator for extension forces as per tension sensor recordings, tension sensor, cable guides, and quick disconnect connector. High gain and clinical performance were achieved by persons affected by stroke using assistive gloves.

To help persons affected by stroke to recover their ability to walk, an assistive control method limb exoskeleton was proposed by Spencer et al. (42). The device worked without modifications with spatiotemporal characteristics of the joint motion. Even after a single session, improvements were visible in patients who used the device with modifications to the gait. Aaron et al. (43) designed a hand extension robot orthosis glove to assist hand-impaired stroke survivors. To extend and flex the fingers, along with the actuator, the gloves included tendons. Inertial measurement device signal thresholds automated the grasp assistance and the robot's finger extension. The glove comprised of a linear actuator, cable tie pawl, battery, microcontroller, selection buttons for manual and automated mode, cable tie tendons, battling glove, open palm, and finger thimbles. The robot orthosis glove provided promising results in terms of spasticity, tone range of motion, pinch, grip, block, box test, hand activity inventory, and Chedoke arm test.

Wing et al. (44) proposed a brain-computer interface to help stroke-affected people with motor ailments. Electrode manufacturing time was minimized with improved reliability and accuracy of 90%. Support vector machine (SVM) recursive feature elimination and fisher's criterion were used for the evaluation of the approach (45). Fisher's approach identified the relationship between the class label and features. Support vectors of the two classes were identified by SVM. For fewer channels, the performance index reduced exponentially. Muscular hypertonus and spasticity were seen in stroke-affected patients affecting their functional characteristics (46). Davide et al. studied the effects of visual feedback and training on arm stiffness while in assisted motion after a stroke attack. The objectives of the study were to get reliable values for the mechanical characteristics of the upper limb while in robotic therapy, acquire visual feedback on viscosity, and identification of arm stiffness effects to examine if the stiffness, training, and visual feedback depended on the level of assistance, speed, and position.

### 2.8. Mobility impairments—Wheelchair, prosthesis

**Figure 6:**
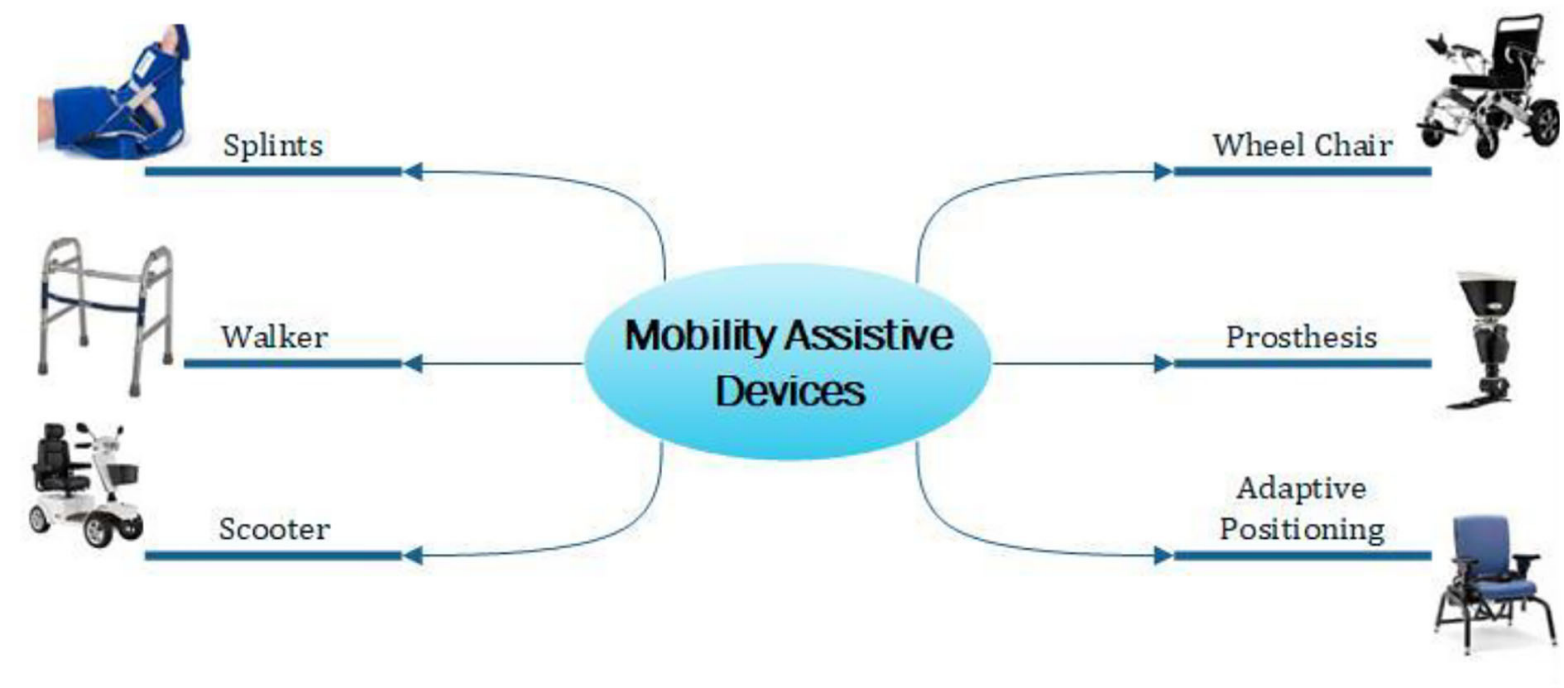
Mobility assistive devices.

Impaired body structures minimize mobility tasks in individuals which may be due to cerebral vascular accidents, limb or spinal cord injuries, multiple sclerosis, or other effects. Assistive technology for the improvement of their movement may be through devices like prosthetic limb control, peripheral nervous system boundary, computer vision improved control, kinematic, dynamic control, and mechanical exoskeletons. Figure 6 shows the mobility-related assistive devices.

Due to motor and cognitive impairments, powering a wheelchair is a tough task for motion-impaired individuals. Mobility assistive devices help individuals to move as per their wish. Automatic wheelchairs with wireless control facilities, intelligent robotics integrated electric wheelchairs, hand rim ergonomics, sit and stand wheelchairs, and illuminated and self-driving wheelchairs are some of the modern wheelchairs that have been developed. Richard et al. (47) proposed an automatic adaptation in the NavChair assistive wheelchair with a control system. Depending on the wheelchair's location and state, the suitable operating mode is chosen and operates through that. However, additional information needs to be provided to the Bayesian network for its improved tasking. Wearable vibrotactile haptics was used for powering the wheelchair in a study by Louise et al. (48). The navigation data was provided by four uniformly spaced vibrotactile armbands. The adaptive arm band reduced collision by 49%.

Emily et al. (49) designed an electromyographically controlled ankle-foot prosthesis to help with rock climbing. Subtalar joints and motorized ankle helped in free space motion. A robotic and traditional prosthesis measured the hip, ankle, and foot joint angles. Subtalar positions and ankle range were improved significantly, reducing maximum knee flexion and hip flexion angles by the proposed approach. The design specifications were 250 mm build height, 1,292 g mass, ±0.008 rad accuracy, 2.18 rad/s velocity, 0.55 Nm free space torque, and 100+ Kg max payload.

A tongue-controlled robotic arm prosthesis was proposed by Johansen et al. (50). The system proved to be efficient by 1.15 s compared to the standard EMG type, with an added feature of computer and environment control. Li et al. (51) proposed a gait evaluation and simulation device for hip disarticulation prosthesis testing. The human motion trajectory was adapted by the robot which had prosthetic thigh simulation. By training an adaptive neural network, the walking pattern was made possible. The neural network solved the big trajectory tracking mistakes and unstable movement. The tested prosthesis kinetic and kinematic data were collected for analysis.

**Figure 7:**
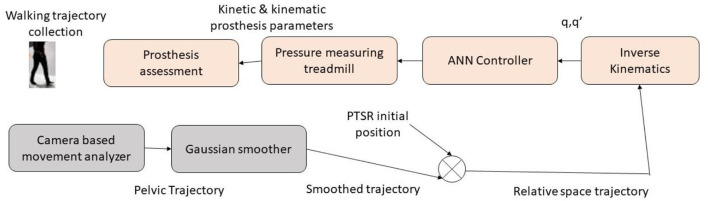
Gait simulation and evaluation device.

The gait prosthesis device architecture can be seen in Figure 7. The Gaussian smoother used the data from the collected trajectory data. The data is mapped into a Prosthetic Thigh Simulation Robot (PTSR). The sensors are connected to the Hip Disarticulation Prostheses (HDP) and PTSR. The smoothed trajectory is mapped to the PTSR's initial position. The ground reaction forces are measured with a pressure-monitoring treadmill. The stability is controlled by an Adaptive Neural Network using the joints database.

### 2.9. Visual impairments

**Figure 8:**
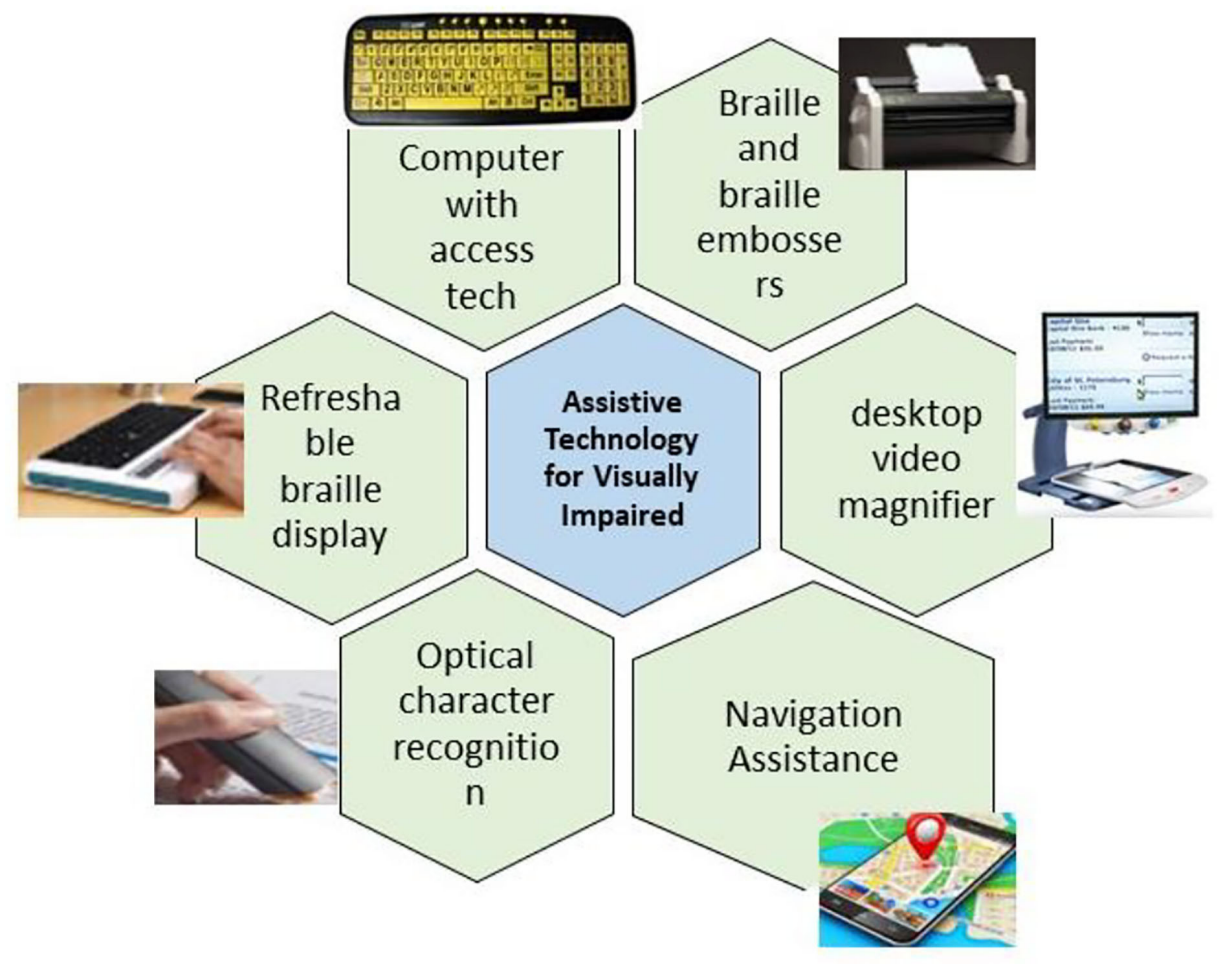
Assistive devices for visually challenged individuals.

The assistive devices which may be applied for visually challenged individuals are tactile huge print keyboards, braille embossers, displays with braille, magnifiers, optical character recognition system, navigation assistance, wearable technologies, canes, screen readers, etc. These devices help individuals live independently by performing their day-to-day tasks. Figure 8 lists some of the assistive devices for the visually challenged.

A haptic telepresence system with depth, color camera, telepresence robot, and haptic interface was designed for visually challenged users to explore rich observation centers by Chung et al. (52). Three-dimensional real-time spatial data access was achieved in the form of point clouds. Through the haptic interface and rendering, the individual can feel the 3D space and control the remotely available robot. By calculating the nearest surface distance and volume interpreting force, the visual proxy force feedback is obtained with a 3D map. Content adaptive multimedia handling and a better sophisticated haptic sensation would improve the system's efficiency.

In the study by Barros et al. (53), educational robotics—CardBot 2.0 is proposed for programming teaching aids. On a table or board, the geometric cards are organized by the individual programming the robot. The teacher can include new languages or tasks for the student. Luis et al. (54) proposed a time-of-flight sensor for a book reader system integrated with a high-resolution CCD camera. The page curvature was corrected by integrating a high-resolution image and the low-resolution time of the flight sensor. The images in the book were flattened and the accuracy of reading was improved by a mathematical framework. 95% reading accuracy was achieved on reading 200 pages. The design setup included a heatsink, bracer, Argos3D-P100 sensor, a book holder platform, and a Canon G6 camera. The acquired images were matched, registered, corrected, optical character recognized, and forwarded to the text-to-speech unit.

### 2.10. Personal emergency response systems

The general personal emergency response system consists of a telephone line connected to a console, a radio transmitter, and an emergency response center. The device is compact and wearable. Voice dialers, touch-and-talk voice pendants, fall detectors, mobile emergency alert systems, and panic button help dialers are some examples of personal emergency response systems. Dahl et al. (55) studied a sensor-based therapeutic observant device that acts as an individual's alternative device with a caregiver view. Fall detection and user-initiated alerts were initiated by the device. The parameters checked were sensor trust, accuracy, ergonomics, form factor, user control, and system feedback.

### 2.11. Augmentative and alternative communication (AAC)

AAC is a communication means used instead of speech or writing for language and speech-challenged individuals. The AAC forms include aided (external support), unaided (facial expressions, gestures), low-tech, and high-tech AAC. its users include intellectually-challenged individuals, persons with autism, cerebral palsy, aphasia, traumatic brain injury, developmental verbal dyspraxia, locked-in syndrome, Parkinson's disease, amyotrophic lateral sclerosis, dementia, and multiple sclerosis.

Gemma et al. (56) presented an AAC device using communication boards and an electronic communicator's speech. The blocks communicate wirelessly. Based on the user's vocabulary and availability of symbols, the communication sheets were designed to be simple, cost-efficient, and with the added advantage of scalability. The sounds were played and recorded by the active sheets in the digital system. Tablets, computers, or smartphones can replace the digital system, providing improved efficiency. The communication sheets are A4 paper classifiers. The grouped messages belong to bigger boxes. Communication devices and electronic controls are powered by a battery source. The sheets provide data to the player through wireless means to get audio output.

Murat et al. (57) provided a brain-computer interface for AAC in clinical and technical domains. The input modalities to the interface include auditory, and visual event-related tasks, volitional cortical tasks, and steady-state evoked tasks. The factors that affect the speller's performance are inter-symbol interval, oddball effect, various matrices, flash organizations, gaze-based, error-related tasks, feature attention, and context information. The physiological input modality used was non-invasive EEG signals. A device with input as voice and outcome as a voice was developed by Mark et al. (58) for AAC to produce synthetic speech from distorted speech. The main device requirements were formulated from the developmental user-centered design. With less available training data, speaker-based automatic speech recognizers with mini vocabularies were built. Even perpetual data provided 96% accuracy performance. The trade-offs were in message production speed and data outcomes range. Geoffrey et al. (59) developed a silent speech recognition system AAC using the surface electromyographic motion of speech musculature from the face and neck and generated speech or speech-to-text forms. The silent speech was also predictable using this approach. A 10.3%-word error rate was achieved.

### 2.12. Older adults

**Figure 9:**
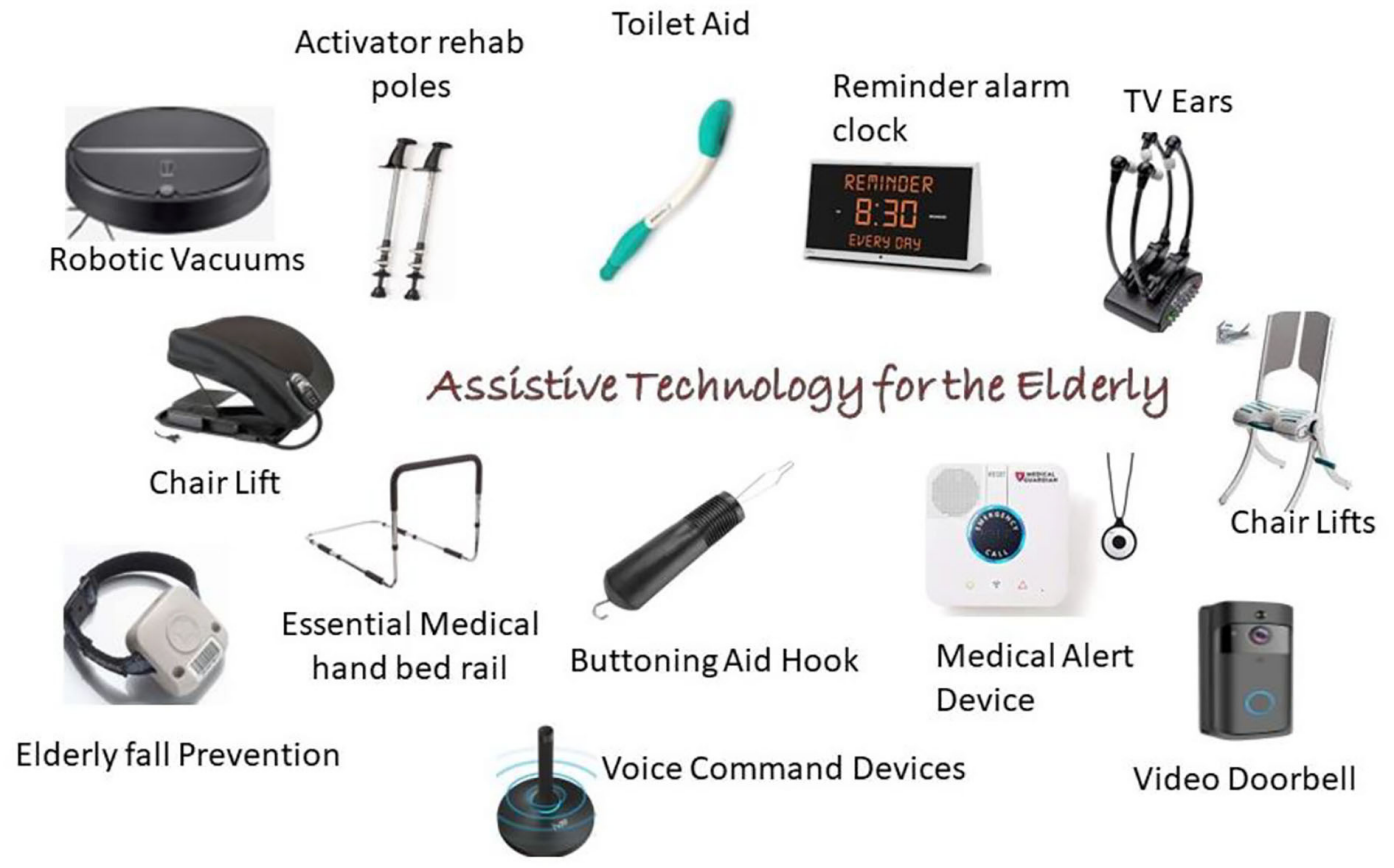
Assistive devices for older adults.

The assistive devices for older adults include medication reminders, telehealth systems, pill dispensers, personal alarms, GPS trackers, communication aids, etc. All these devices are user-dependent and based on their knowledge to handle and their minor requirements. The devices focus primarily on mobility, self-care, safety, and communication (Figure 9).

The study by Ester et al. (60) discusses assistive technology used by older adults such as robot assistants and physical and cognitive rehabilitation for older adults. Physical rehabilitation, speech therapy, social assistants, healthcare companion robots, and many similar such assistive technologies have been discussed by the authors. In their study, Juan et al. (61) propose a socially assistive robot-human interaction to encourage older adults to exercise regularly. High user preference was seen in using this product. Workout, imitation, and memory games were accessible in the system. Feng et al. (62) discuss using an ambient intelligent device for older adults using home appliances. The device consisted of activity identification, sensor synthesizing, and case-dependent reasoning. Depending on the sensors available in the smart home, rough set theory was used. Improvement in context consciousness was visible. Context identification included layer recognition, context middleware layer, sensing, and perception layer. Followed by context modeling, case comprehension included assisting, controlling, and acting layers leading to assistance action.

### 2.13. Hearing impairments

Hearing loss influences access to spoken language, involving cognition and evolution, and negatively impacts social wellbeing. Auditory impairment can affect the quality of life and spoken communication, inhibiting the evolution of a child's spoken language, and presenting the risk of dementia and cognitive deterioration in older ages (63).

Chung et al. used a sound-sensitive adaptive directional microphone responding to one direction alone. The sound location was found with high volume and improvements in SNR (64). Best et al. used pinna effect simulation—a multi-channel adaptive directional microphone designed to revive the impact of pinna for behind-the-ear type aids. Filtering was not performed, and the hearing aid received the input audio (65).

Keidser et al. (66) studied the effects of directional microphones, minimizing noise in hearing aids. The authors reviewed available data separately from the front, back, left, and right dimensions and concluded that the performance during horizontal localization was the most changed out of all the signal-related parameters. The front and back errors were minimized and right and left errors increased on applying various microphones. The localization sense may be upgraded for hearing aid-using individuals.

### 2.14. Cognitive impairments—Educational software

The use of educational tools for the literacy process of differently-abled individuals has received great attention from the Special Education field from the point of view of inclusive education. This field has encouraged methods and technology created to promote learning through assistive technologies. However, most of the software and hardware which can embed programming for the differently-abled was not much successful.

Lucas et al. designed a digital game for learning-deprived children. The system consisted of education methods based on Paulo Freire's techniques. The games were developed and implemented for differently-abled children. These games include continuous monitoring and analysis of the understanding and practice levels addressed in the games. The successful adoption of the proposed technology showed acceptable results (67).

Chaita et al. implemented LIBRAS educational software in Portuguese for hearing-challenged students in their schools. The creative and exciting software included digital games that enable students to understand more recent and adequate means of communication (68).

Acuna et al. investigated the construction of the torso of a humanoid robot built by an additive manufacturing process with an interactive graphical user interface developed for students to self-learn the language. The humanoid was assessed with students using traditional teaching and a humanoid robot. With the humanoid robot, the students could recite the self-learning process many times and learning times were decreased by 25%. Analogized to the traditional method, it operated as a trainer and served as an interactive means of contact between the hearing-challenged community and listeners (69).

In another study, Aranyanak et al. proposed a device that tracked the reader's finger movements in real time based on an Android tablet.

It was equipped with:

- Online visual knowledge on finger movement practices.
- Total reading duration.
- Average reading pace.

This permitted investigators to analyze braille reading in better profundity to enhance the readers' braille reading dexterities (70).

## 3. Inferences

Based on the discussions in the previous sections, the following findings and inferences are being made:

(i) Differently-abled individuals who need assistive devices prefer to perform their daily tasks on their own despite the availability of devices that perform the same task. Artificial intelligence-based technology can help customize the devices to meet the specific needs of individuals.
(ii) The study population in most of the studies is very less, which makes it difficult to project device usability on a larger scale.
(iii) Assistive devices have drastically reduced the burden on the caregiver and formal health and support services. More research in this field will help individuals improve their social interaction, opportunities, and functioning.
(iv) Economic and cost-benefit analyses of available devices are not discussed by the researchers, which is an essential factor in the usage of rehabilitation devices.
(v) Caregiver injury and the individual user's injury in the absence of a caregiver have not been studied. A detailed study of the pros and cons of the assistive device is essential for its usage and further development.

An overview of the year of publication of recent research on assistive technology reviewed in this paper is listed in Table 2.

**Table 2:** Year of publication of recent state of art research papers on different assistive technology fields.

**Dementia**	**Autism**	**Knee replacement**	**Spinal chord injury**	**Exoskeleton**	**Diabetes-therapeutic footwear**	**Stroke **	**Mobility impairments— wheel chair, prosthesis**	**Visual impairments**	**Personal emergency response system**	**Augmentative and alternative communication (AAC)**	**Elderly**	**Hearing impairments**	**Cognitive impairments —educational software**
2022	2020	2021	2021	2022	2017	2022	2021	2022	2021	2022	2022	2022	2019
2021	2019	2020	2016	2021	2016	2020	2017	2021	2017	2021	2021	2021	2015
2020	2016	2018	2015	2020	2015	2019	2016	2020	2016	2020	2020	2020	2014

Most of the reviewed research on assistive devices is marked by the rapid development of the field, yet, at the same time, there are gaps and scope for further research.

## 4. Discussion

This section discusses additional challenges and future scope in the usage of assistive and rehabilitative devices.

### 4.1. Challenges

A comprehensive review of current literature on the subject has flagged multiple challenges.

(i) The limited study population and the support provided to the individual and the caregiver are important factors in analyzing the usefulness of the technology. The sample population is very less in most studies although the requirement for the devices is significantly huge.
(ii) Economic assessments of the effectiveness of assistive technology are not reported in the studies relating to impairment assistive device evaluation.
(iii) Identification and awareness of the appropriate device, its quality, and its use, along with the assistive device's access, availability, and adaptability are the present challenges in its wider and regular use.
(iv) Standardized protocols are required for different assistive technology designs and those that need them with a common lexicon.
(v) Device effectiveness is mostly dependent on surveys, which may be biased because of users' enthusiasm and unstructured feedback.
(vi) It is imperative to develop multidisciplinary devices that are flexible and based on user requirements.
(vii) The involvement of the caregiver and family makes a difference in device usage.
(viii) Lack of training, skills, and knowledge of device usage may lead to improper usage causing inconvenience to the user.
(ix) User attitude toward assistive devices and self-training are the drawbacks of assistive device usage.

### 4.2. Future scope

In this review, efforts have been taken to highlight new technologies and devices that address the needs of differently-abled individuals and older adults. A few potential avenues for further research which can extend the field of assistive technology have been mentioned below.

(i) Current research in the field of assistive technology uses exploratory designs strategy based on current knowledge levels. Exploratory research based on hypothesis will help in getting optimum caregiver response.
(ii) Qualitative methods are necessary to analyze the assistive devices.
(iii) A study of other unseen factors that may impact using a device will improve assistive device adaptability for individuals.
(iv) Artificial intelligence and machine learning-based assistive devices can be developed to improve the usability and flexibility of the devices.

The future for potential research in assistive technology development is unlimited. Hardware and software development are required to bring affordable assistive devices to a wider population that needs them.

## 5. Conclusion

A comprehensive discussion on various assistive technology applications and devices has been undertaken in this study. Assistive technology is a multi-disciplinary research field with huge research gaps and immense potential. The choice of specific assistive technology depends on the task to be performed, social influence, the type of technology, and the individual's choice. This paper reviewed the research in assistive technology which may be adopted for the improvement of the individual's day-to-day tasks. From this study, it is evident that there is a need for further research and development in the field of assistive technology which is likely to expand tremendously in the future. Better integrated devices reduce the human burden and enable differently-abled individuals to lead self-sufficient lives. Intelligent and accurate computer-based devices are the need of the hour to address the needs of differently-abled individuals and older adults.

## Author contributions

PM performed conceptualization, methodology, design, data collection, data visualization, formal analysis, reviewing, and editing. YT carried out conceptualization, methodology, design, investigation, data collection, data analysis, and writing original draft preparation. SL done conceptualization, methodology, data collection, data visualization, formal analysis, reviewing, and editing. SD involved in data curation, critical analysis, writing, reviewing, and editing. KL contributed in formal analysis, reviewing, and editing. XW involved in reviewing and editing. All authors contributed to the article and approved the submitted version.
